# Subbrow Lift Using Frontalis Sling to Correct Lateral Orbital Laxity

**DOI:** 10.1007/s00266-020-01879-9

**Published:** 2020-07-24

**Authors:** Hong Seok Kim, Kenneth K. Kim

**Affiliations:** 1 Ohkims Plastic Surgery Clinic, Hyosan Building, 5th Floor, 861 Janghang-dong, Ilsan-dong-gu, Gokyang city, Kyung-gi-do South Korea; 2 UCLA School of Medicine, Los Angeles, CA USA

**Keywords:** Blepharoplasty, Subbrow, Frontalis, Aging eyelids

## Abstract

**Background:**

In order to correct upper lid laxity, upper blepharoplasty, subbrow excision, and forehead lift have been utilized. Our newly developed subbrow excision attaches the orbicularis oculi muscle to the frontalis muscle. This improves the longevity of the result without inhibiting the gliding plane of the periorbita.

**Method:**

From January 2016 to July 2018, 564 patients were operated on using this technique. Among them, 41 were male and 523 were female with the average age of 59.5 years. The average size of the subbrow excision was 55 mm × 8 mm. From the upper skin incision site, the upper dissection proceeded cephalad in the subcutaneous plane just above the orbicularis oculi muscle to the point where the frontalis muscle was seen. The lower flap was created by incising the orbicularis oculi muscle 5 mm cephalad to the distal skin incision. From this 5-mm orbicularis muscle stump, the dissection proceeded caudally in a plane between the orbicularis muscle and the orbital septum. Once this flap was created, the 5-mm muscle stump was attached to the exposed frontalis muscle in a horizontal mattress fashion in three areas. The skin incision was then closed. Three months after the operation, a satisfaction survey was conducted using the Likert scale.

**Results:**

The patients were followed postoperatively for at least 6 months. In all but two cases, the orbital laxity improved. However, in the brow’s lateral third where the frontalis muscle does not exist, a slight lowering of the brow had occurred. The incision healed well without any keloid or hypertrophic scars. There were no significant complications such as superior orbital nerve entrapment-related sensory problems.

**Conclusions:**

Subbrow lift utilizing the frontalis muscle attachment to the lower flap orbicularis muscle is a novel method of correcting upper eyelid skin hooding. The technique does not rely on periosteal fixation. Therefore, the eyebrow gliding plane is not violated. Thus, the natural eyebrow movement is maintained. There were no cases of injury to the deep branch of the supraorbital nerve, poor wound healing, or other significant complications.

**Level of Evidence IV:**

This journal requires that authors assign a level of evidence to each article. For a full description of these Evidence-Based Medicine ratings, please refer to the Table of Contents or the online Instructions to Authors www.springer.com/00266.

## Introduction

**Fig. 1:**
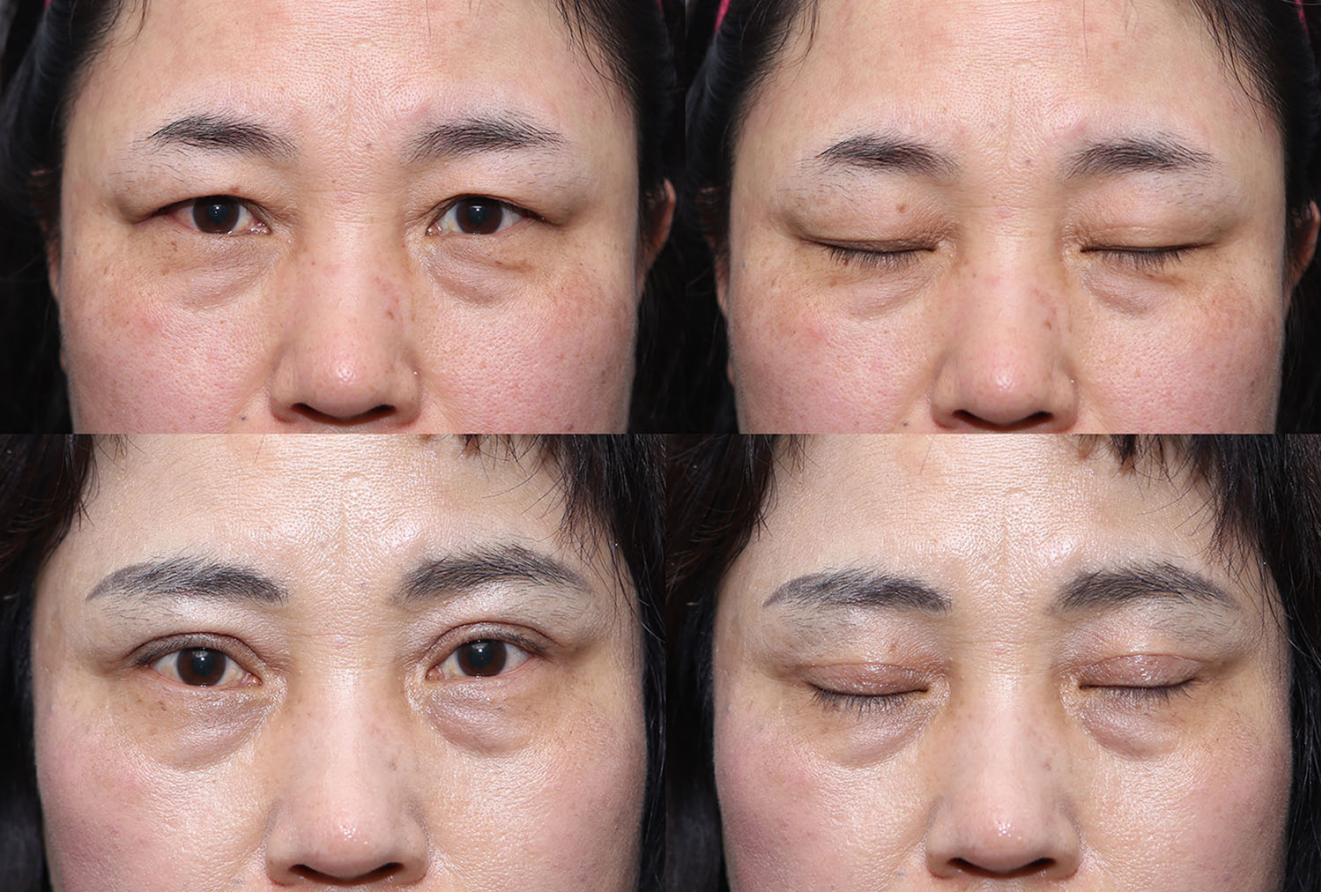
Picture of standard upper blepharoplasty in patients with brow ptosis. (**a** preoperative photograph, **b** postoperative follow-up 6 months. The attachment of the supratarsal fold to the preorbital skin creates an unnatural, harsh, and puffy appearance)

The first part of the face that manifests aging is the periorbital region [1,2]. There are various methods to correct the sagging eyelid and hooding of the lateral upper eyelid/subbrow region [3,4]. The simplest method is to excise the upper eyelid skin via upper blepharoplasty [5]. However, during the upper blepharoplasty excision, most of the pretarsal skin and sometimes even the preseptal skin get excised. Therefore, the supratarsal fold becomes attached to the thicker preorbital skin without the intermediate skin thickness transition. This leads to an unnatural appearance (Fig. 1). In addition, because the skin incision is carried out laterally to excise the lateral skin, the incision becomes noticeable.

**Fig. 2:**
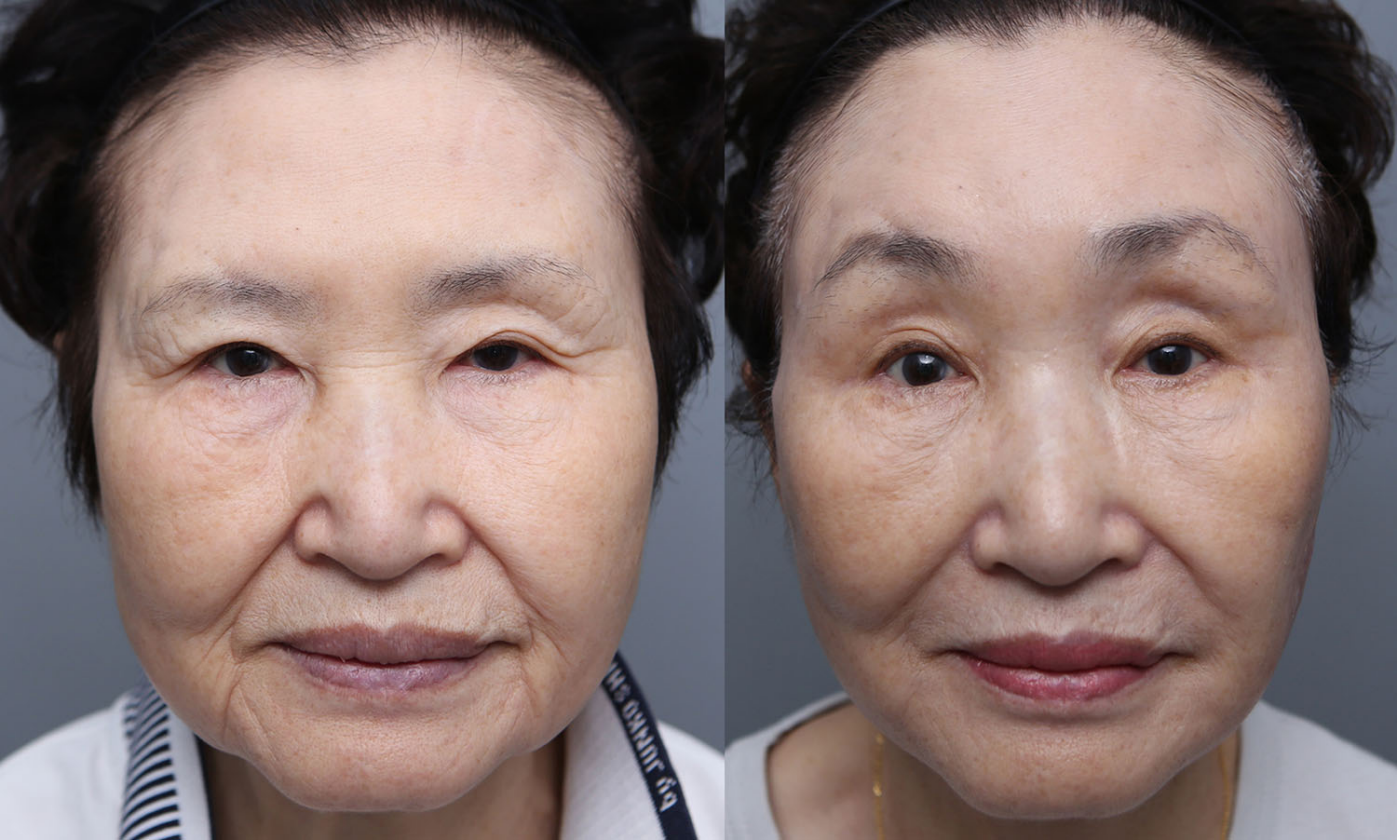
Picture of eyebrow over-elevation (left: preoperative photograph, right: postoperative. Endoscopic brow lift can over-elevate the brows producing a surprised appearance.)

Endoscopic brow lift or coronal brow lift can be a viable solution for clearing sagging eyelid skin [6,7]. However, in cases where there is minimal brow ptosis, endoscopic brow lift or coronal brow lift can over-elevate the brow and thus give a surprised look (Fig. 2). In this type of high or normal brow position, subbrow excision can correct the orbital laxity without making the brows and eyelids appear unnatural.

In previously described subbrow excision techniques, the skin is simply excised [8–10] or the orbicularis oculi muscle is excised and the lower flap is fixated to the periosteum [11,12]. The simple skin excision is prone to early recurrence. The fixation to the periosteum method may appear effective initially, but it too is subject to relapse and even brow ptosis. This is because the lower flap fixation to the periosteum has to pass through the gliding plane of the brow movement which ultimately loosens with constant brow movement.

Our technique attached the orbicularis oculi muscle to the distal end of the frontalis muscle. Therefore, the fixation was superficial to the gliding plane. Thus, the fixation did not impede brow movement and corrected the eyelid skin laxity.

## Patients and Methods

From January 2016 to July 2018, 564 patients underwent the subbrow excision technique. There were 41 males and 523 females. The ages ranged from 25 to 75 years with the average age of 59.5 years. The average excision amount was 55 mm × 8 mm. There were 354 patients who underwent simultaneous double eyelid fold surgery without skin excision (Fig. 7).

### Design

**Fig. 3:**
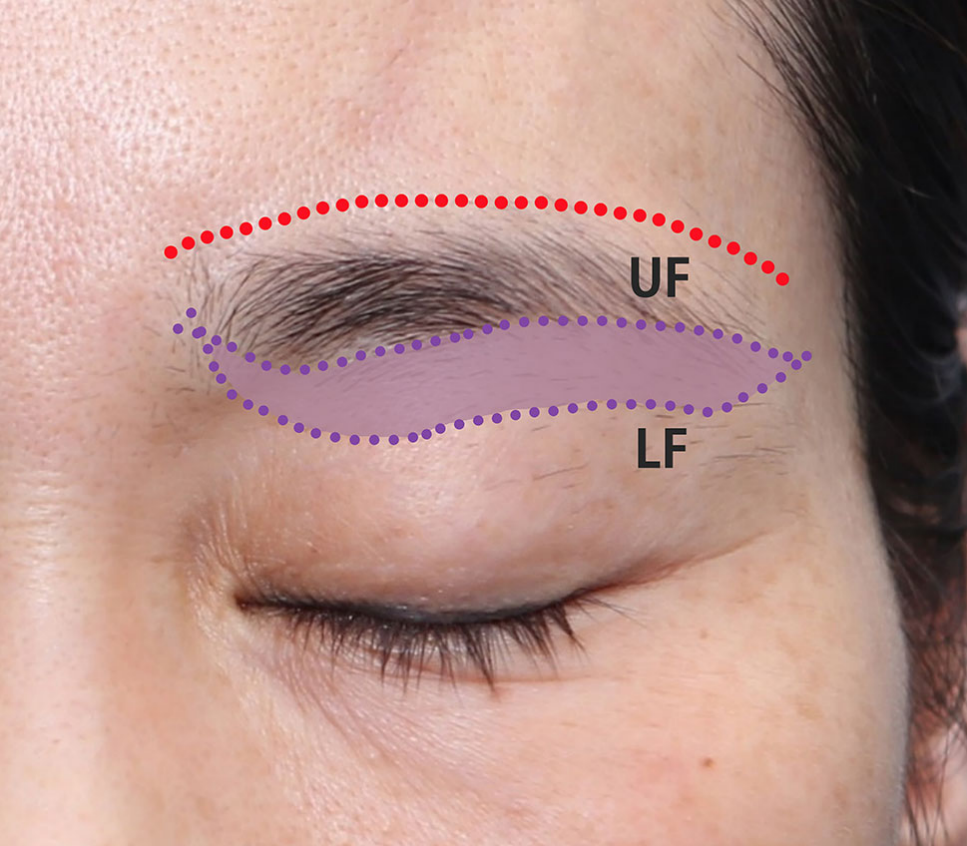
Picture of subbrow marking. The lower and upper incision marks were tapered at the corners to prevent dog ear formation. (UF: upper flap, LF: lower flap; the red dotted line is the dissection limit. The purple area is the range to remove the skin.)

The upper incision was marked along the inferior margin of the brow. The lateral distance was based on the amount of lateral brow laxity. In cases of significant brow ptosis, care was made to not make the lateral incision below the supraorbital ridge. The lower incision was made by lifting the brow and marking the amount of skin that would clear the hooding eyelid skin. The subbrow mark to the lower mark was the amount of skin that needed to be excised. The lower incision mark was parallel to the upper incision mark and was tapered at the corners to prevent dog ear formation (Fig. 3).

### Operative Methods

Surgery was done with local anesthesia (lidocaine 1% with 1:100,000 epinephrine) with or without IV sedation. Following the marked designed of the subbrow, the skin excision was done with a 15 blade. This exposed the orbicularis oculi muscle. From the upper incision site, the cephalic dissection was made between the skin and the orbicularis oculi muscle for about 15 mm using electrocautery. At the supraorbital ridge region, the transverse muscle fiber of orbicularis oculi and the vertical muscle fiber of frontalis transition were seen. However, at the lateral third of the region, lack of frontalis muscle was noted.

From the lower incision line, a mark was made 5 mm cephalad with a marking pen. Then, the caudal dissection was made by cutting through the orbicularis oculi muscle at the marked line with a Bovie and then dissecting caudally. This left a stump of orbicularis oculi muscle. The caudal dissection was at the plane between the orbicularis oculi muscle and the orbital septum for 7–8 mm.

**Fig. 4:**
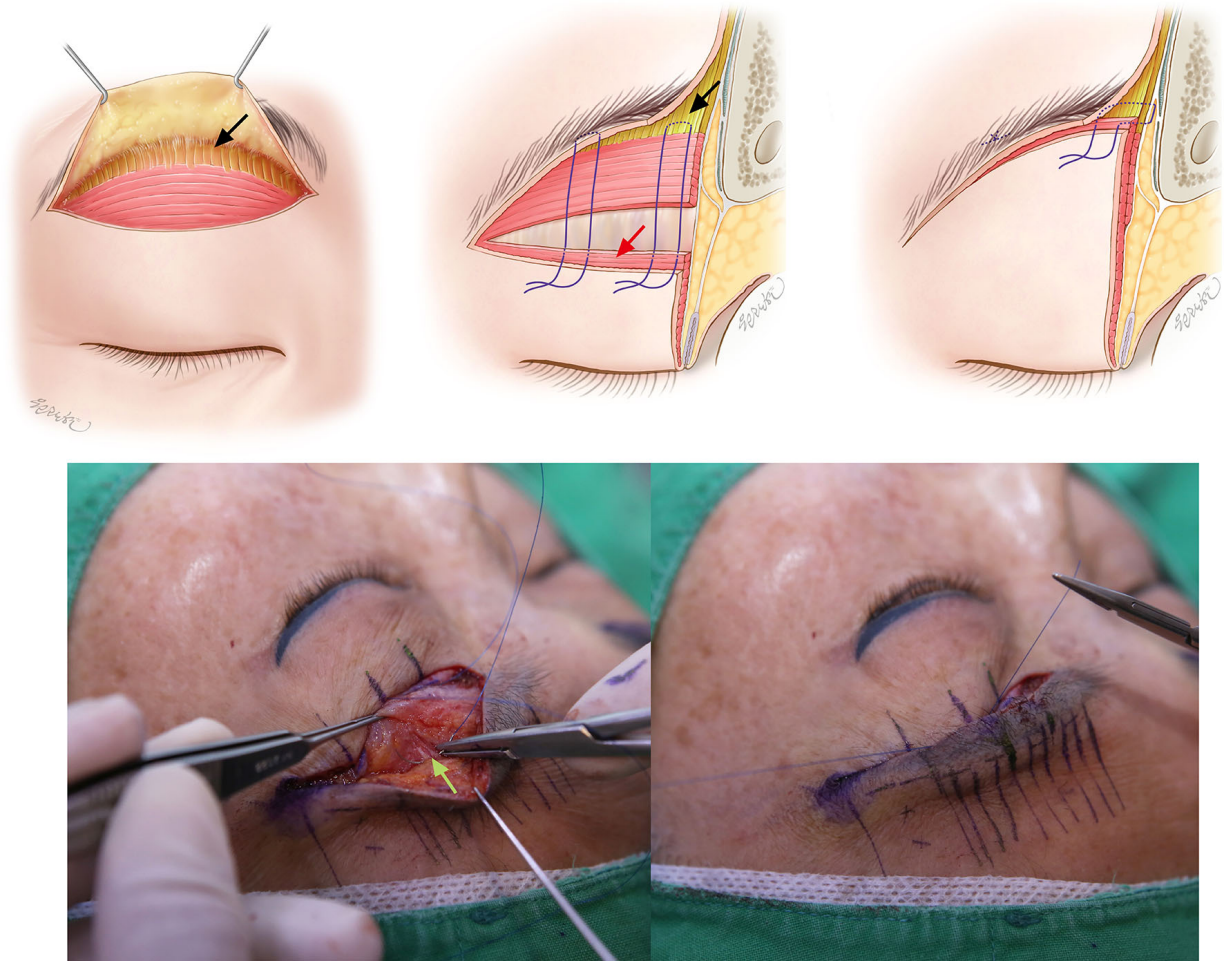
Picture of frontalis sling fixation. From the lower flap orbicularis oculi muscle stump margin, sutures were applied to the exposed frontalis muscle in 3 areas in a horizontal mattress fashion. (**a** schematics of frontalis sling, black arrow: frontalis, red arrow: orbicularis oculi muscle stump of lower flap, **b** intraoperative photograph, green arrow: distal area of frontalis, **c** intraoperative photograph, after frontalis sling fixation suture)

The remaining orbicularis muscle from the stump to the upper skin incision site was excised to minimize upper brow bulk. From the lower flap orbicularis oculi muscle stump margin, three-point fixation sutures were applied to the distal end of the frontalis muscle with 5–0 Prolene sutures in a horizontal mattress fashion (Fig. 4). At the lateral fixation region where the frontalis muscle ends, the suture was carefully made to purchase the lateral end of the frontalis muscle. Prior to the subcutaneous suture placement, the lower flap dermis was gently undermined by 1–2 mm to level the skin edges. At the dermal edges, 10–15 buried 5–0 Vicryl sutures were placed to approximate the subcutaneous and skin edges. Multiple 7–0 nylon sutures were then placed in a continuous fashion to close the skin. The sutures were removed 5–7 days later, and the healing process was observed for 6 months postoperatively.

For patients who wished to double fold, a double fold operation was performed by incision method without excision of the skin. We performed double fold surgery using a method of incising the skin and the orbicularis oculi muscle and fixing the lower flap to the SAJT (septoaponeurotic junctional thickening).

## Results

**Fig. 5:**
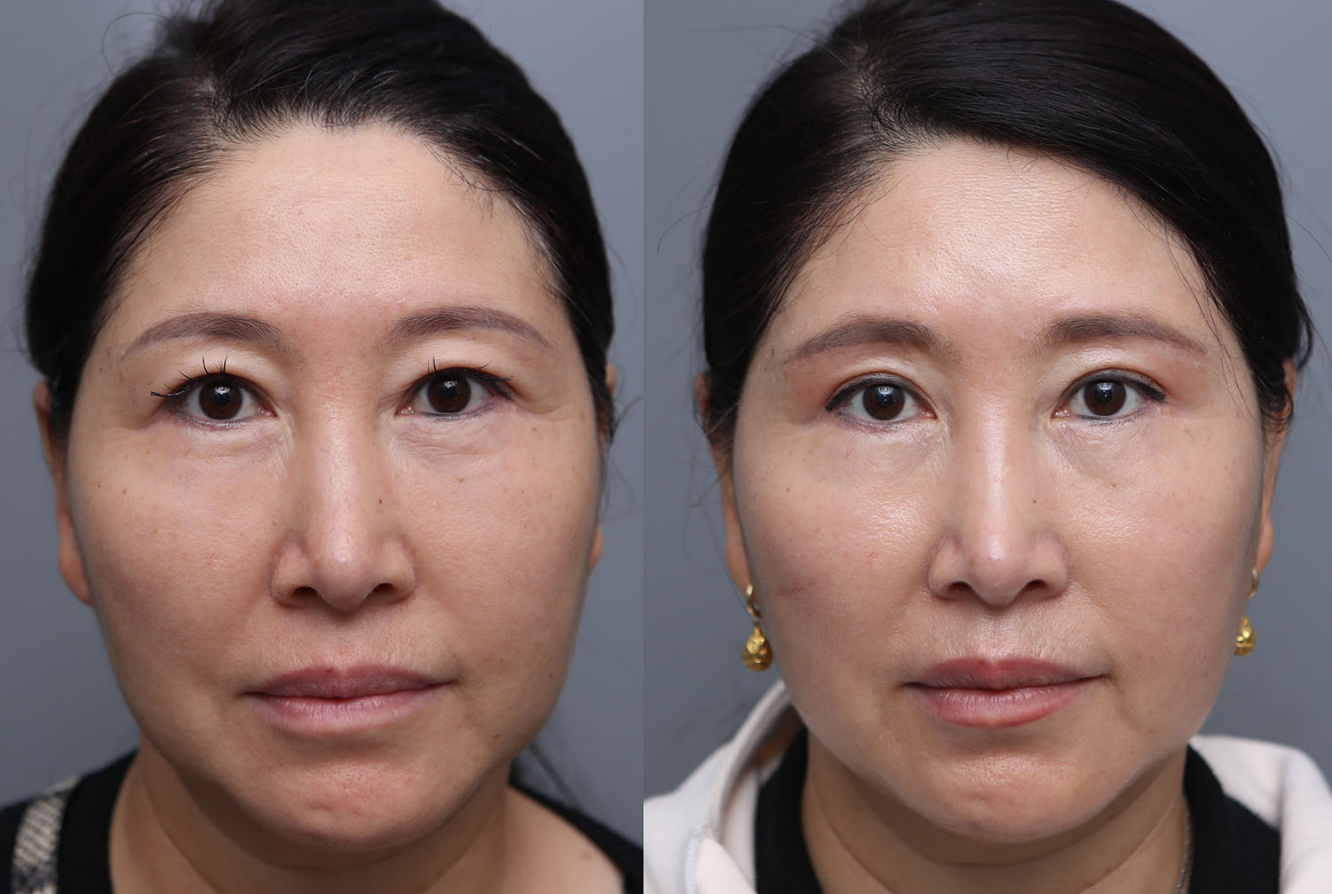
Picture of before and after of patient 6 months after subbrow excision utilizing frontalis sling. (**a** Preoperative photograph **b** Postoperative 6 months)

**Fig. 6:**
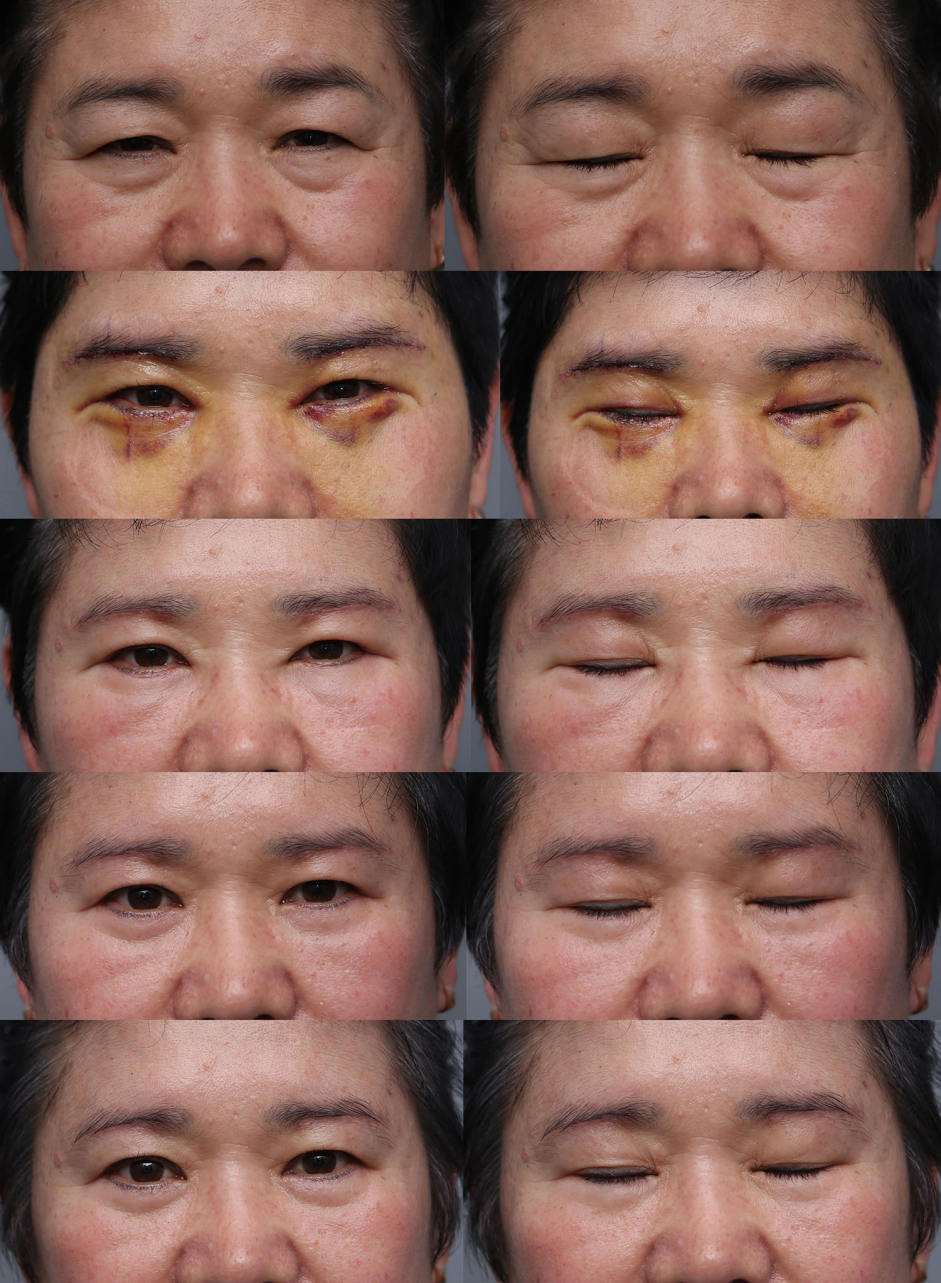
Picture of before and after of patient 6 months after subbrow excision utilizing frontalis sling. (Patient underwent subbrow excision and lower lid blepharoplasty. **a** preoperative photograph, **b** postoperative follow-up 5 days, **c** postoperative follow-up 3 weeks, **d** postoperative follow-up 3 months, **e** postoperative follow-up 6 months)

**Fig. 7:**
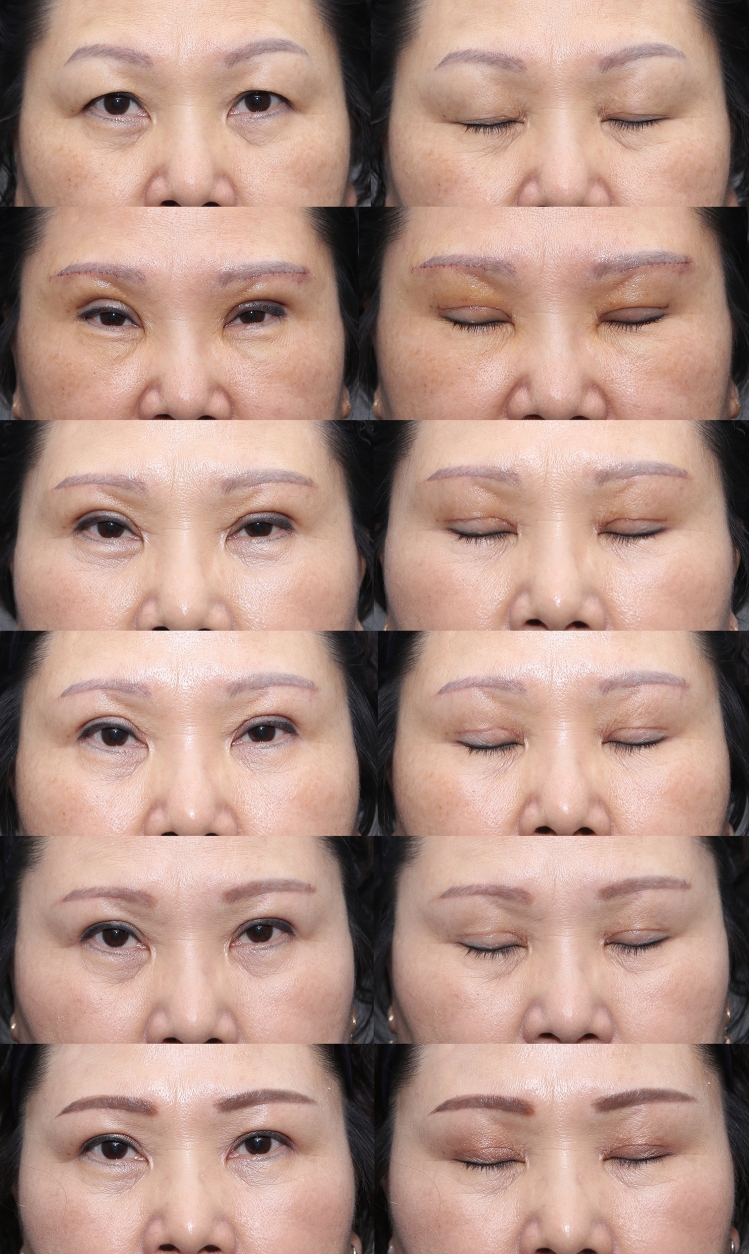
Picture of before and after of patient 6 months after subbrow excision utilizing frontalis sling. ( Patient underwent subbrow excision with double fold surgery, **a** preoperative photograph, **b** postoperative follow-up 6 days, **c** postoperative follow-up 3 weeks, **d** postoperative follow-up 7 weeks, **e** postoperative follow-up 12 weeks), **f** postoperative follow-up 9 months)

Postoperatively, the incision and the healing process were observed at 3 weeks, 2 months, 3 months, and 6 months. Immediately after surgery, bunching of the incision site was noted. However, within 3 weeks, the bunching at the incision site had disappeared. All of the redness and scar elevation had disappeared within 3 months. There were no symptoms of supraorbital nerve trapping throughout the entire recovery process. Improvement of the lateral orbital laxity was noted, and the inferior margin of the eyebrow incision site healed well by the 6-month period (Figs. 5, 6, 7). At the lateral third of the subbrow region where there is no frontalis muscle, a slight descent of the brow was noted. There were 5 cases of temporary incision numbness, 11 cases of under correction, 1 case of stitch abscess, and 1 case of tattoo related inflammation.

The Likert scale was used to determine the efficacy of the procedure in correcting upper orbital laxity (Table 1). Out of 564 patients, 437 patients participated in the survey. There were 15 patients who did not notice an improvement or only slight improvement. There were 422 patients (97% of surveyed) who noticed somewhat improved, much improved, or markedly improved results.

**Table 1:** The following are the patient survey results from the Likert scale (total 437 patients answered at 3 months after surgery)

Degree of satisfaction after surgery (Likert scale)		Number of patients
1	Not improved	2
2	Slightly improved	13
3	Somewhat improved	72
4	Much improved	249
5	Markedly improved	101

## Discussion

There are many causes of periorbital regional soft tissue laxity and ptosis. Gravity and tissue degeneration from aging cause the lateral brow to descend and the skin to lose elasticity. In addition, patients with senile blepharoptosis compensate their weak eyelid muscles by raising their forehead muscles. This cephalic movement of the forehead and brow further stretches the lateral brow skin. In addition, the relaxation of the frontalis muscle upon rest causes subsequent brow descent. This upward and downward movement of the brow over a prolonged period of time induces brow laxity and brow ptosis.

Excising skin at the brow region is an excellent option to improve lateral orbital laxity. Excision of the skin above the brow (suprabrow excision) can often lead to visible scars. Therefore, subbrow excision has gained popularity in Asia. Among the subbrow excision techniques, there have been two main types of subbrow excisions. The first method is excision of the subbrow skin only [10]. The other method is fixating the lower flap to the upper orbital periosteum [12,13]. Although the excision of the skin technique is simple, it leads to early recurrence and somewhat noticeable scarring due to tension. The other subbrow method of fixating the lower flap skin muscle to the upper orbital periosteum by YS Kim [11] lasts longer than the simple subbrow skin excision technique. This periosteal fixation method may initially appear stable due to firm attachment. However, this fixation violates the brow gliding plane which is the extension of the galea to the brow. As the patient makes upper facial expressions and raises the brows, the brows move along this gliding plane. Therefore, this frequent movement in the gliding plane ultimately loosens the periosteal attachment and the brows descend. In addition, because the deep branch of the supraorbital nerve is positioned at the upper third of the brow, the periosteal fixation can entrap the supraorbital nerve or cause neuropraxia. For these reasons, we have not performed either a skin excision only or fixating to the periosteum subbrow techniques for many years. Therefore, we did not have a control group of either skin excision only or fixating to the periosteum in our study.

Our method does not violate the gliding plane of the eyebrow. Our dissection and fixation method all reside at a submuscular layer. Our technique connects the orbicularis muscle to the insertion end of the frontalis muscle. Therefore, there is no restriction in the deeper gliding plane region, leading to a natural brow movement. In addition, our method leads to less chance of recurrent brow ptosis.

The deep branch of the supraorbital nerve penetrates the periosteum and traverses cephalad in a plane between the galea aponeurotica and the pericranium to supply sensation to the frontoparietal scalp [14]. Since our dissection plane was more superficial and the fixation was to the distal frontalis muscle, the chance of nerve entrapment from the fixation was minimal as noted in our results. The superficial branch of the supraorbital nerve penetrates through the frontalis muscle to supply the forehead and the anterior region of the scalp. Therefore, during the dissection and fixation, care is needed to prevent injury to the superficial branch. It is worthwhile to note that injury to the deep branch of the supraorbital nerve rather than the superficial branch, causes most distress, numbness, and paresthesia.

There are many versatilities in our technique compared to the conventional subbrow lift techniques. In cases of sunken upper eyelids, the dissected lower flap allows clear visualization for micro-fat grafting to be placed at the retroorbital oculi fat (ROOF), within the orbital septal layers/orbital septal membrane. On the contrary, in cases of puffy or fatty upper eyelids, the lower flap dissection allows removal of fat from the ROOF or from the orbital septum. Our method, subbrow lift using frontalis sling, is equally applicable to non-Asian patients. Since non-Asians tend to have thinner orbital tissue (less weight), better results in terms of aesthetics, quality of the scar, and longevity can be expected after the surgery.

In patients with glabella frown lines, our technique allows easy access for resection of the corrugator and procerus muscle. Because our subbrow technique’s upper medial dissection resides near these muscles, a clear direct visualization of the frowning muscles allows for muscle resection with minimal neurovascular injury.

Compared to the conventional subbrow lift techniques, our lower flap dissection and the subsequent advancement to the forehead muscle, minimizes tension during closure. Therefore, we did not see any scar widening or hypertrophic scars in our series. For the first 3–6 months following surgery, redness of the scar was noticed. But after 6 months, the redness disappeared and all of our patients were satisfied with the overall scar and wound healing process.

There were no significant complications from our technique. We noticed some temporary numbness at the incisional site. However, all incisional numbness in our series resolved within 3 to 6 months. In our series, there were no patients with permanent numbness or nerve injury.

## Conclusion

Our subbrow technique involves wide bidirectional dissection to minimize tension upon closure and utilizes a frontalis sling mechanism to correct upper orbital laxity. Rather than a static fixation to the periosteum, the frontalis muscle suspension of the orbital muscle does not violate the brow gliding plane. Therefore, the surgery restores functionality and the natural facial expressions of the forehead/brow/eyelid complex. We saw a marked improvement of the orbital laxity without injuring the superior orbital nerve deep branch. In addition, we observed satisfactory wound incision healing without scar widening.
